# Promoting Consciousness Recovery in Patients with Acquired Brain Injury Through Multimodal Sensory Stimulation: A Randomized Controlled Trial of Median Nerve and Auricular Stimulation

**DOI:** 10.1007/s12028-026-02543-x

**Published:** 2026-05-28

**Authors:** Hai Li, Wei Song, Shuping Zeng, Ling Liu, Changyu Liu, Hanjing Lu

**Affiliations:** 1 Department of Rehabilitation Medicine, The First Affiliated Hospital of Gannan Medical University, Ganzhou, 341000 China; 2 Department of Rehabilitation Medicine, Shanghai Fourth People’s Hospital, School of Medicine, Tongji University, Shanghai, 200434 China; 3 Department of Rehabilitation Medicine, The First People’s Hospital of Nankang, Ganzhou City, 341411 China; 4 Department of Gastroenterology, The First School of Clinical Medicine, Gannan Medical University, Ganzhou, 341000 China; 5 Department of Anesthesiology, The First School of Clinical Medicine, Gannan Medical University, Ganzhou, 341000 China; 6 Department of Emergency Medicine, The First Affiliated Hospital of Gannan Medical University, Ganzhou, 341000 China; 7 Key Laboratory of Emergency and Trauma of Ministry of Education, Key Laboratory of Hainan Trauma and Disaster Rescue, Department of Emergency Medicine, The First Affiliated Hospital, Hainan Medical University, Haikou, 570102 China

**Keywords:** Disorders of consciousness, Median nerve electrical stimulation (MNES), Auricular intradermal needle, Acquired brain injury (ABI)

## Abstract

**Background:**

Addressing disorders of consciousness (DOC) secondary to acquired brain injury (ABI) remains challenging in neurorehabilitation. Median nerve electrical stimulation (MNES) is a noninvasive therapeutic option, but its benefits as monotherapy are restricted. The auricular intradermal needle, a prolonged-stimulation traditional Chinese medicine technique, has not been assessed for consciousness restoration. This study examined the safety and effectiveness of combining MNES with auricular needling for patients with DOC post-ABI.

**Methods:**

This randomized controlled trial enrolled 116 patients with DOC (GCS 4–8), randomly assigned to a treatment group (*n* = 58, standard care + MNES + auricular intradermal needling) or a comparison group (*n* = 58, standard care + MNES). MNES was administered twice daily for 60 min per session; auricular needles were embedded for 3 days (three pressings/day), with the total protocol spanning 11 days. Primary outcome was Coma Recovery Scale-Revised (CRS-R) score, evaluated at 4 weeks postintervention and 3 months post-ABI. Secondary outcomes included GCS score, awakening rate, Glasgow Outcome Scale (GOS) score, and family satisfaction.

**Results:**

Intent-to-treat (ITT) analysis showed that the experimental group had a significantly greater CRS-R score increase than controls at 4 weeks (mean difference: 1.62, *P* = 0.022) and 3 months (mean difference: 2.05, *P* = 0.006). All secondary outcomes differed significantly: higher GCS scores, higher awakening rates (48.28% vs. 27.59% at 4 weeks; 62.07% vs. 41.38% at 3 months), more favorable GOS outcomes (4–5: 55.17% vs. 31.03%), and improved family satisfaction. No serious treatment-related adverse events occurred.

**Conclusions:**

Integrating MNES with auricular intradermal needle enhances therapeutic outcomes for DOC following ABI, proving more effective than MNES alone in promoting consciousness recovery and neurological improvement without compromising safety. This complementary method represents a viable and promising advancement in noninvasive DOC management.

*Trial registration* ClinicalTrials.gov Identifier: NCT 07273864.

**Supplementary Information:**

The online version contains supplementary material available at https://doi.org/10.1007/s12028-026-02543-x.

## Introduction

Disorders of consciousness (DOC) are a spectrum of clinical syndromes, defined by a significantly impaired or complete lack of awareness of external stimuli, primarily owing to damage to or disruption of neural circuits that regulate wakefulness and conscious awareness [1, 2]. The causative agents of DOC are typically traumatic brain injury (TBI) alongside ischemic and hemorrhagic stroke, hypoxic–ischemic encephalopathy, and various other severe neurological insults [3]. Globally, TBI is experienced by more than 50 million people annually [4], some of whom might develop long-term disorders of consciousness, among them the vegetative state (VS), also known as the unresponsive wakefulness syndrome (UWS) and the minimally conscious state (MCS). These patients experience considerably reduced quality of life, and their management inflicts an enormous care and financial burden on families and society [5].

In Gannan, a district of Jiangxi Province in China, the projected permanent population is 8.96 million by early 2025. The high rate of diseases associated with consciousness disorders, including TBI, is influenced by the high frequency of traffic accidents and the presence of some residents that work in high-risk industries in the area, which has resulted in a significant burden on healthcare resources of the First Affiliated Hospital of Gannan Medical University annually. TBI is still a leading cause of death and disability among young adults globally [6]. Low- and middle-income regions are especially challenged in providing effective prehospital emergency services, surgical, and rehabilitative care [7]. Thus, the elaboration and testing of an efficient, safe, and conveniently adopted consciousness-promoting intervention are essential in enhancing the neurological outcome of patients receiving DOC and reducing the family and societal cost.

It is important to recognize that disorders of consciousness are not solely disorders of arousal, most patients have widespread structural brain injury affecting multiple functional networks. Therefore, while promoting the return of conscious awareness is a critical early goal, the ultimate therapeutic objective extends beyond consciousness restoration to encompass long-term functional recovery and reduction of disability burden. Consciousness recovery serves as a foundational step that enables subsequent neurorehabilitative interventions to enhance neuroplasticity and improve functional outcomes. This conceptual framework positions our multimodal stimulation strategy within a broader rehabilitation pathway for ABI-related DOC.

Although pharmacotherapy, hyperbaric oxygen, sensory stimulation, and neuromodulation are current DOC management modalities [8], their efficacy remains controversial [9, 10]. For example, amantadine has been shown to accelerate functional recovery in patients with traumatic DOC [11, 12], but its efficacy in nontraumatic cases remains uncertain [13], and it is associated with side effects [14]. Invasive methods such as deep brain stimulation (DBS) and spinal cord stimulation are faced with limitations owing to their complexity, risks, and limited applicability [15]. Therefore, the focus of the current research is the establishment of evidence-based, noninvasive, and easily applicable neuromodulation methods.

The median nerve serves as a vital peripheral pathway that ensures a wide connectivity to the central nervous system [16]. Over the last 20 years, median nerve electrical stimulation (MNES) has been proven as a noninvasive neuromodulatory method with potential to manage DOC [17, 18]. Clinical studies [19, 20] and meta-analyses [21] have provided evidence that MNES can increase the level of consciousness and reduce the number of days in a coma among DOC patients. However, the effects of MNES as a monotherapy are limited, and the prolonged treatment duration restricts its clinical applicability.

**Fig. 1:**
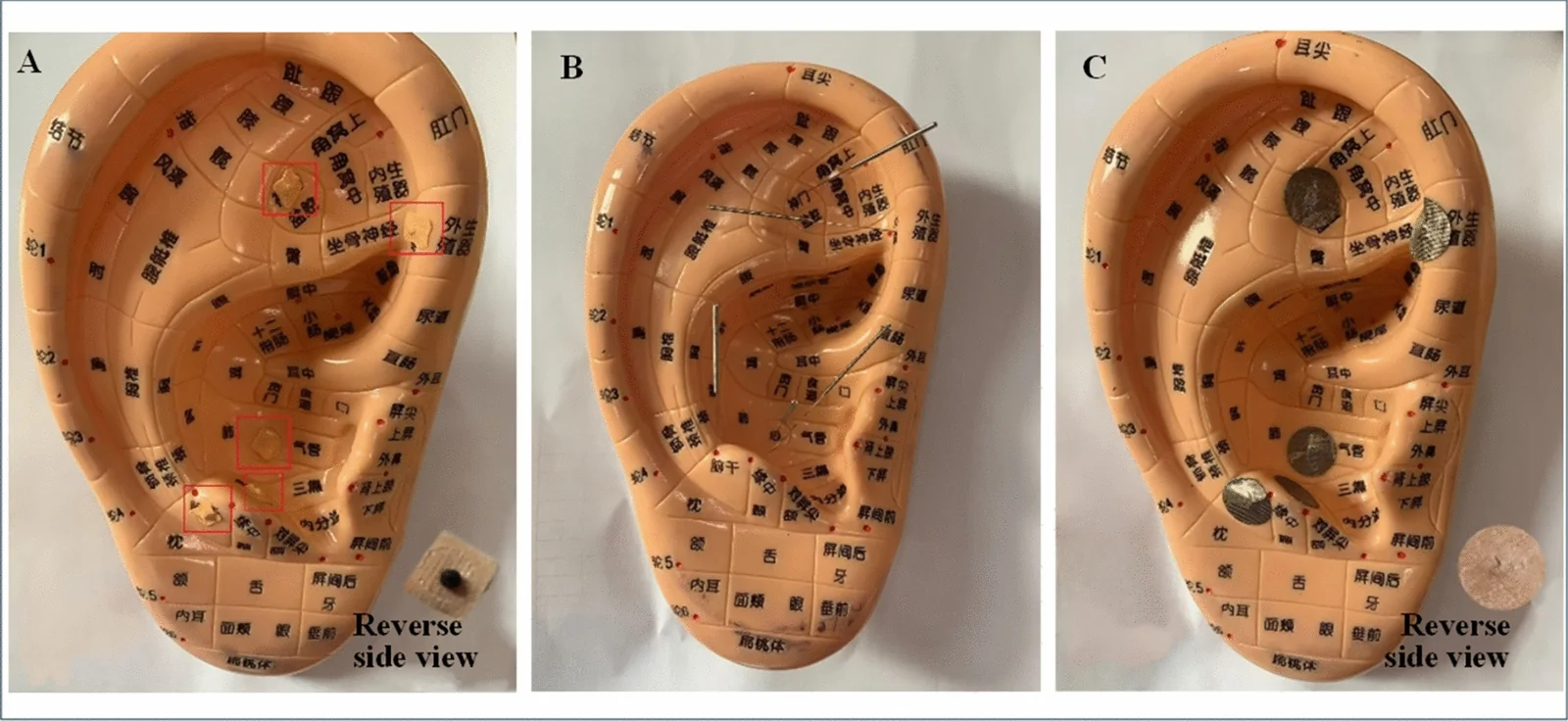
Schematic diagram of different auricular therapy devices. **A** Auricular acupressure using Vaccaria seeds. **B** Auricular acupuncture with filiform needles. **C** Auricular intradermal needle for short-term embedding

The ear in traditional Chinese medicine (TCM) is regarded as a microcosm of the body with the vessels of the ancestors accumulating there and in turn linking to internal organs by meridians. Auricular acupuncture can regulate physiological processes and establish overall balance by stimulating certain points in the ear [22, 23]. Auricular therapy is a traditional Chinese treatment which involves the use of auricular acupressure and auricular acupuncture. Auricular acupressure: Intermittent manual stimulation of an auricular point with special equipment, e.g. Vaccaria seeds (Fig. 1A), or fine needles into an auricular point (Fig. 1B). The somatotopic theory of auricular points was proposed by the “father of auricular acupuncture,” Paul Nogier, in 1956, thus contributing to the current medical concept of traditional auricular therapy. The method employed in this study (Fig. 1C) is the “auricular intradermal needle” technique, which uses small, embedded needles to provide continuous mild stimulation over multiple days. This approach is easy to use, well-tolerated, and can overcome the limitation of MNES’s short stimulation duration. Despite the benefits of auricular therapy in the management of pain, insomnia, and the regulation of autonomic nervous system [22, 24, 25], its opportunities in the facilitation of consciousness recovery have not been properly explored and deserve further study.

While both MNES and auricular stimulation may involve modulation of vagal-related pathways, they differ substantially in their neural entry points, stimulation characteristics, and network-level effects. MNES acts primarily via the median nerve to activate the ascending reticular activating system (ARAS) through the spinal nerve–reticular formation–thalamus–cortex pathway [26, 27], providing a strong, intermittent, and phasic neuromodulatory stimulus that broadly enhances cortical excitability and arousal [28–30]. In contrast, auricular intradermal needle stimulation targets the auricular branch of the vagus nerve (ABVN) and trigeminal nerve afferents, which project to the nucleus tractus solitarius (NTS) and subsequently modulate brainstem nuclei (e.g., locus coeruleus) and cortical networks involved in awareness, including the default mode network and prefrontal cortex [31, 32]. This provides a milder, continuous, and tonic neuromodulatory input. Therefore, rather than representing two forms of the same vagal stimulation, these interventions engage partially overlapping but functionally distinct neural circuits—MNES targets broad arousal networks, while auricular stimulation influences autonomic, limbic, and higher-order cognitive networks. Their combined use is hypothesized to produce complementary effects by providing both phasic and tonic neuromodulation at multiple levels of the neural hierarchy, thereby enhancing neuroplasticity and facilitating consciousness recovery more effectively than either alone.

Currently, there is no research that has directly evaluated the therapeutic efficacy of the integration of MNES with the use of an auricular intradermal needle therapy on patients with DOC after ABI. To mitigate this existing research deficit, we conducted a randomized controlled trial (RCT) to evaluate whether this multimodal sensory stimulation approach can enhance consciousness recovery in patients with DOC following ABI. We aim to develop a safe and effective integrated rehabilitation protocol that facilitates the return of conscious awareness, a critical first step toward long-term functional recovery, while also contributing to the integration of Chinese and Western medical practices.

## Methods

### Study Design and Participants

This was a prospective randomized controlled trial testing the safety and efficacy of bilateral MNES and auricular intradermal needle to assist in consciousness restoration in patients with brain injury with DOC. The trial was conducted throughout the intensive care unit (ICU) and rehabilitation department of the First Affiliated Hospital of Gannan Medical University, between June 2022 and September 2025. This study protocol was approved by the hospital Ethics Committee (Approval number: LLSL-2022053105) and adhered to all the ethical standards stipulated by the Declaration of Helsinki. Trial registration: ClinicalTrials.gov Identifier: NCT 07273864. Informed consent was obtained in writing on behalf of all enrolled patients by statutory family members after full disclosure on the procedures involved in the study, including possible risks and benefits. The design and outcome reporting of this trial were in compliance with the CONSORT guidelines.

A comprehensive clinical evaluation of all participants was conducted before enrollment and fulfilled the following inclusion criteria: (1) DOC due to traumatic brain injury, brain hemorrhage, cerebral infarction, or hypoxic–ischemic encephalopathy with clinical manifestations such as coma, unresponsive wakefulness syndrome, or minimally conscious state; (2) a Glasgow Coma Scale (GCS) score ranging from 4 to 8 at the time of enrollment; (3) stable vital signs, with no uncontrolled intracranial hypertension or active bleeding; (4) within 7–14 days following the onset of brain injury^]^ [33]; (5) aged between 18 and 75 years; (6) written informed consent voluntarily provided by a legally authorized family member.. Major exclusion criteria were: (1) severe systemic diseases (e.g., heart, liver, or kidney failure); (2) a history of epilepsy, major psychiatric disorders, or cognitive impairments that could affect treatment evaluation; (3) pregnancy or breastfeeding; (4) known allergies to the electrodes or materials used in the study; (5) participation in other interventional clinical trials simultaneously; (6) failure of the patient’s family to cooperate with treatment or withdrawal from the study during the trial. In cases of serious adverse events, complications, or disease worsening during the trial, the trial would be discontinued immediately according to the decision of the physician. Clinical management would be offered immediately in case of termination of the trial depending on the symptoms. After terminating the trial, the physician needs to investigate the case, record the causes, observe data, and interpolate the efficacy and adverse reactions data based on the final test results.

Overall, 116 qualifying patients were enrolled and were randomized to a reference group or an intervention group with 58 participants each. This research aimed at enrolling a mixed population that is heterogeneous in age, gender, and the cause of brain damage to enhance the research findings generalizability. The comparative analysis of baseline demographic and clinical data showed that there were no statistically significant differences between the two groups, and the profiles were well balanced, thus reducing the possibility of confounding effects of treatment results.

### Randomization and Blinding

A rigorous randomization, allocation concealment and blinding design was adopted to give the study objectivity and scientific rigor. Eligible participants were randomly assigned to either experimental or control group in a 1:1 ratio according to a computer-generated randomization, with allocation concealment implemented via sequentially numbered, opaque sealed envelopes. The random sequence was generated by an independent statistician, and envelopes were only opened after participant enrollment to confirm group assignment. After randomization, patients were not informed of their group assignment during the treatment, intervention, and follow-up phases. Moreover, group assignments were not disclosed to all outcome assessors in data collection and data evaluation stages. The blinding design of this study reduced the influence of subjective variables on the outcomes and avoided the effect of psychological expectations on the outcomes of the treatment in the analysis by patients or researchers. Also, in a data analysis stage, statisticians did not know the group assignments, which again guaranteed the objectivity of the analysis process.

### Interventions

Every patient enrolled in the study underwent standard comprehensive treatment, in addition to the assigned interventions. The holistic therapy involved a vital check, respiratory and circulatory support, symptom pharmacotherapy, and early rehabilitation care, which meant that all patients were given the same baseline treatment to compare with. On this basis, the two groups were given the following interventions:

The control group received standard intervention of bilateral MNES. The stimulation was delivered using a multifunctional electrotherapy device (Model: MT1023, Shenzhen Dongdixin Technology Co., Ltd.). Surface electrodes were placed on the ventral face of each forearm, about 2 cm above the distal wrist crease, to stimulate the median nerve. The stimulation program used a direct current asymmetric square wave whose parameters were as follows: the output current was 20 mA, pulse width was 300 ms, and frequency set at 40 Hz. The stimulation was administered in a cyclic manner, which included 20 s of active stimulation, separated by rest intervals of 40 s. Each session lasted 60 min, and MNES was administered twice daily (morning and afternoon) with a minimum of 4 h between sessions (the actual interval was 6–8 h in clinical practice, consistent with the morning-afternoon schedule). The treatment was continued at 7 days per week until a total treatment period of 4 weeks.

**Fig. 2:**
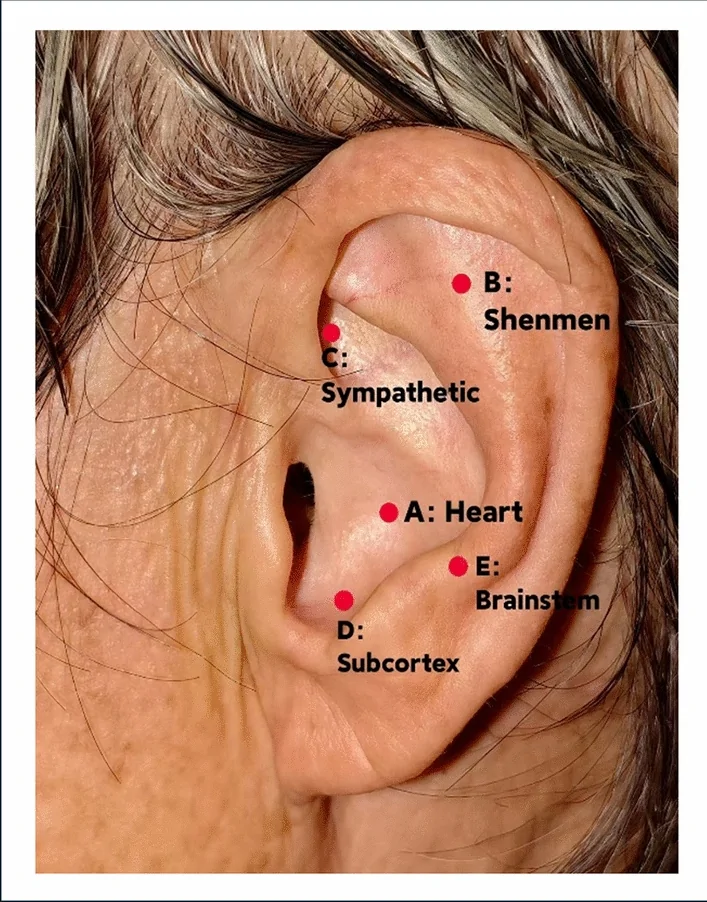
Distribution of auricular points treated with auricular intradermal needle in the experimental group, including **A** Heart (CO15), **B** Shenmen (TF4), **C** Sympathetic (AH6), **D** Subcortex (AT4), and **E** Brainstem (AT3/4i)

The experimental group patients received an addition of auricular intradermal needle therapy to the conventional comprehensive treatment and bilateral MNES protocol. The needles were disposable sterile press needles (Model: 0.20 × 0.8 mm) manufactured by Suzhou Medical Supplies Factory Co., Ltd. The acupoints chosen were the “Heart,” “Shenmen,” “Sympathetic,” “Subcortical,” and “Brainstem” points on both ears (Fig. 2). These points were selected on the basis of a dual rationale. From the perspective of TCM, these points are classically indicated for regulating mental activities, calming the spirit, and restoring autonomic balance. From a neuroanatomical standpoint, several of these points are located within areas innervated by the auricular branch of the vagus nerve and trigeminal nerve, which provide afferent input to brainstem nuclei involved in arousal regulation (e.g., nucleus tractus solitarius, locus coeruleus) and project to cortical networks associated with awareness. This was done by trained rehabilitation therapists. Following the routine disinfection of the ears, the press needles were implanted and secured into position. After needle insertion, participants or caregivers were told to perform manual pressure on the embedded needles three times a day over 10 per session. Press needles were retained for 3 days and then changed, and the same part of the ear was allowed 1 day rest before retreatment. They repeated this cycle three times and the overall intervention lasted 11 days.

### Outcome Measures

The primary outcome measure was the Coma Recovery Scale-Revised (CRS-R), a standardized neurobehavioral assessment tool specifically designed for patients with disorders of consciousness [34, 35]. The CRS-R was selected as the primary end point owing to its well-validated psychometric properties and its sensitivity in detecting subtle signs of awareness, enabling reliable differentiation between VS/UWS and MCS, a critical diagnostic distinction in this patient population. All patients underwent CRS-R assessment at baseline (at enrollment, prior to intervention, 7–14 days postinjury), at 4 weeks postintervention, and at 3 months post-ABI.

Secondary outcome measures included: (1) the GCS score [36], assessed at baseline (enrollment), at 4 weeks postintervention and at 3 months post-ABI to reflect overall changes in the patient’s conscious state; (2) the recovery rate as the proportion of patients who emerged from VS/UWS to MCS or higher based on CRS-R diagnostic criteria—specifically, demonstration of command following, intelligible verbalization, object recognition, or visual pursuit, based on subscale item performance rather than total score. The recovery rate was assessed at 4 weeks and 3 months post-ABI; (3) the Glasgow Outcome Scale (GOS) score [37, 38], assessed at 3-month follow-up. GOS classifies patients according to independence in function and restoration to everyday living, where scores of 1 (death), 2 (vegetative state), and 3 (severe disability) denote unfavorable functional outcomes, whereas scores of 4 (moderate disability) and 5 (good recovery) indicate favorable functional outcomes; (4) satisfaction levels among families, assessed at 3 months following ABI through a 5-point Likert scale (spanning from 1, very dissatisfied, to 5, very satisfied) for evaluating perceptions regarding therapeutic effectiveness from relatives and caregivers of patients. For ensuring data quality, all assessments were conducted by qualified personnel following standardized operational protocols.

To ensure the validity and reliability of assessments, all evaluations were conducted by trained clinicians experienced in DOC assessment, following standardized operational protocols. All assessors were licensed rehabilitation physicians or neurologists with at least 3 years of clinical experience in managing patients with disorders of consciousness. They received formal training in the administration of the CRS-R, GCS, and GOS, including hands-on practice and inter-rater reliability assessments prior to the study. The standardized operational protocols were developed on the basis of published guidelines and manuals for each scale, detailing patient positioning, stimulus presentation, and scoring criteria to ensure consistency across assessors and time points. Assessments were performed at a consistent time of day (morning) in a quiet environment free from distractions. When patients were under the influence of sedative medications or when arousal level fluctuated, assessments were postponed or repeated within 24 h to obtain the most representative score.

To maintain objectivity, treatment and outcome assessment were performed by separate teams: the treatment team (rehabilitation therapists) delivered the interventions and managed any adverse events or treatment interruptions, while the assessment team (trained clinicians) performed CRS-R and GCS evaluations and were blinded to group allocation. Assessors did not participate in treatment, had no access to treatment records, and conducted assessments in a separate area with patients’ ears covered to conceal the presence of auricular needles. In the event of treatment interruptions or medical complications, these were managed by the treatment team without involvement of the assessors. If accidental unblinding occurred (e.g., patient disclosure), a different blinded assessor performed the reassessment.

### Statistical Analysis

The statistical power calculation in the form of G*Power software (version 3.1.9.7) was used to determine the requisite sample size at the time of planning this trial. The calculation basis was anticipated between-group disparities on the main outcome measure which was the CRS-R score at 3 months post-ABI. According to previous randomized trials that used MNES [3], a moderate effect size (Cohen’s *d* ≈ 0.5) was expected. Independent samples *t*-test formed the analytical basis, with specification of a bilateral alpha level at 0.05 together with 80% statistical power. Such computation indicated that no fewer than 51 participants in each group were needed. Considering anticipated attrition or loss-to-follow-up approximating 10%, enrollment was increased to 58 participants within each group, producing a total planned cohort of 116 individuals. This adjustment was implemented to preserve the study’s statistical robustness for analyzing the primary end point. Ultimately, the final recruited sample aligned with this projection and satisfied the prespecified power requirements.

Statistical computations employed SPSS Statistics version 27.0 from IBM Corp. headquartered in Armonk, NY, USA, alongside GraphPad Prism version 10.3.1 developed by GraphPad Software located in San Diego, CA, USA. Assessment of normality for continuous variables utilized the Shapiro–Wilk test. Data exhibiting normal distribution appear as mean accompanied by standard deviation, whereas variables showing non-normal distribution are shown as median with interquartile range (IQR). Within-group temporal variations, such as from baseline through week 4 and from baseline through 3-month follow-up, underwent evaluation via paired *t*-tests. Between-group contrasts at corresponding temporal points employed independent samples *t*-tests.

Variables of a categorical nature, including awakening rate and sex distribution, were presented using frequencies and percentage values. Intergroup contrasts for these categorical outcomes employed the chi square test, alternatively applying Fisher exact test when anticipated cell frequencies dropped beneath five. A bilateral alpha criterion of *P* < 0.05 governed all statistical evaluations. For preserving the intention-to-treat framework, multiple imputation (MI) was performed using SPSS version 27.0 to handle the single missing outcome, generating 20 imputed datasets, with results pooled according to Rubin’s rules.

### Study Monitoring and Safety

An independent Data and Safety Monitoring Board (DSMB), composed of specialists in intensive care medicine, rehabilitation medicine, and statistics, was tasked with routine oversight and safety evaluations across the trial duration. All enrolled participants received ongoing surveillance of vital physiological indicators, encompassing heart rate, blood pressure, respiratory rate, and peripheral oxygen saturation, with documentation performed daily by trained nursing personnel. Additionally, the research team systematically tracked and reported all adverse events (AEs) occurring during the study period, regardless of their suspected relationship with the intervention. AEs were classified as intervention-related (e.g., localized pain, erythema, discomfort during electrical stimulation, local infections caused by ear acupuncture) or nonintervention-related (e.g., infections, fever, electrolyte imbalances) based on clinical judgment. All AEs were graded according to the Common Terminology Criteria for Adverse Events (CTCAE v5.0), and their seriousness and potential relatedness to the study procedures were assessed by the research team. Between-group comparisons of AE incidence were performed using Fisher’s exact test.

Given that patients with DOC cannot self-report pain, monitoring relied on objective indicators of distress during and after stimulation sessions, including changes in vital signs (heart rate, blood pressure, respiratory rate), facial expression, motor agitation, and autonomic responses. Any abnormal physiological fluctuations or observable signs of discomfort were documented and evaluated by the treating physician. In the event of serious adverse events (SAEs), the relevant intervention would be immediately discontinued, and symptomatic treatment would be provided. These events were promptly reported to the ethics committee and independently evaluated by the DSMB. This trial’s execution maintained strict adherence to standards established within the Declaration of Helsinki and Good Clinical Practice (GCP) guidelines, thereby protecting welfare of participants while securing integrity of the study outcomes.

## Results

**Fig. 3:**
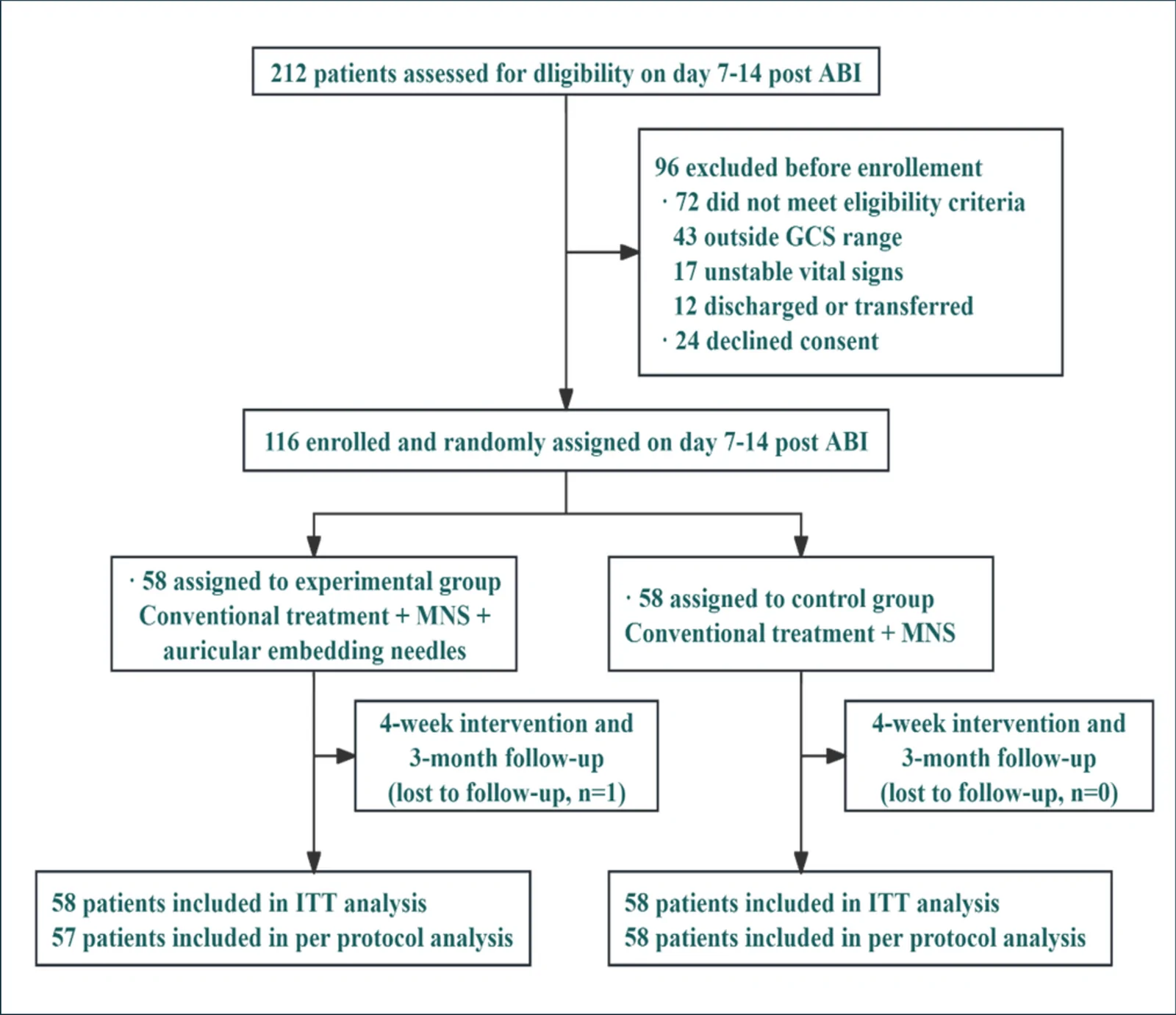
CONSORT flowchart

Between June 2022 and September 2025, screening was performed on 212 consecutive patients for potential inclusion in the study, as outlined in Fig. 3. Following screening, 96 individuals were excluded, with the main reasons being failure to satisfy the predefined inclusion criteria (*n* = 72) or a decision to decline enrollment (*n* = 24). The other 116 qualified subjects were then randomized into two treatment groups. The experimental arm (*n* = 58) received a combination of conventional therapy, MNES, and auricular intradermal needle intervention, whereas the control arm (*n* = 58) was administered conventional therapy alongside MNES. Over the course of the 4-week treatment phase and the ensuing 3-month follow-up period, a single participant in the experimental arm was lost to follow-up. In contrast, all patients allocated to the control group completed the study according to protocol.

Table 1 summarizes the baseline demographic and clinical profiles of the study participants, showing that the two treatment groups were well-balanced at enrollment. The median time from injury to enrolment was 11 days [IQR: 8–12] in the experimental group and 10 days [IQR: 9–11] in the control group, with no significant difference between groups (*P* = 0.652), confirming that the two groups were well-balanced with respect to the postinjury recovery stage at enrollment. All analyses of primary and secondary outcome measures in this study strictly adhered to the ITT principle. Although one participant from the experimental group was lost to follow-up at the final 3-month assessment point, to address this missing data, MI was performed using SPSS version 27.0 (20 imputed datasets, results pooled according to Rubin’s rules). Consequently, the final ITT analysis included the full set of 116 randomized patients, with 58 individuals in each study arm. A supplementary per-protocol (PP) analysis was conducted to assess the consistency of the findings, which incorporated 57 and 58 participants in the experimental and control groups, respectively. The outcomes of this sensitivity analysis were consistent with the primary ITT analysis based on MI and, thus, support the soundness of the study findings.

**Table 1:** Baseline demographics and characteristics of the ITT population

	Experimental (*n* = 58)	Control (*n* = 58)	*P* value
Demographic characteristics			
Age (median [IQR], years)	56 [52, 62]	56 [50, 62]	0.568
Gender			
Male (%)	45 (77.6)	40 (69.0)	0.402
Female (%)	13 (22.4)	18 (31.0)	
Past medical history (Comorbidities)			
Hypertension (%)	37 (63.8)	40 (69.0)	0.695
Diabetes mellitus (%)	7 (12.1)	16 (27.6)	0.061
Coronary heart disease (%)	7 (12.1)	4 (6.9)	0.528
History of cerebrovascular disease (%)	13 (22.4)	13 (22.4)	> 0.999
Other comorbidities (e.g., CKD, COPD) (%)	7 (12.1)	3 (5.2)	0.322
At admission			
ABI causes (%)			
TBI	18 (31.0)	15 (25.9)	0.711
ICH	16 (27.6)	13 (22.4)	
CI	19 (32.8)	22 (37.9)	
HIE	5 (8.6)	8 (13.8)	
Initial GCS (median [IQR])	6 [4, 7]	6 [5, 7]	0.294
Initial pupillary reflex (%)			
Both present	39 (67.2)	42 (72.4)	0.840
One absent	12 (20.7)	10 (17.2)	
Both absent	7 (12.1)	6 (10.3)	
At enrollment (day 7–14 post ABI)			
Enrollment Time (post-injury day, median [IQR])	11 [8, 12]	10 [9, 11]	0.652
Systolic BP (median [IQR], mmHg)	128 [118, 137.3]	132 [122.5, 139.3]	0.099
SpO₂ (median [IQR], %)	97 [96, 99]	97 [95.8, 98.2]	0.451
Pupillary reflex (%)			
Both present	46 (79.3)	49 (84.5)	0.630
One absent	12 (20.7)	9 (15.5)	
Both absent	0 (0)	0 (0)	
GCS (median [IQR])	7 [5, 8]	7 [6, 8]	0.550
CRS-R (median [IQR])	7.5 [5.8, 9]	8 [7, 9.3]	0.462

Continuous measures were expressed as median [IQR], whereas categorical measures were shown as *n* (%). For intergroup contrasts, continuous measures underwent evaluation via Mann–Whitney *U* test, whereas categorical measures were assessed through χ^2^ test or Fisher’s exact test (applied when anticipated frequency fell below 5). Etiological classification of ABI: TBI, traumatic brain injury; ICH, intracerebral hemorrhage; CI, cerebral infarction; HIE, hypoxic–ischemic encephalopathy. Key abbreviations: ABI, acquired brain injury; GCS, Glasgow Coma Scale; CRS-R, Coma Recovery Scale–Revised; BP, blood pressure; SpO₂, peripheral oxygen saturation; CKD, chronic kidney disease; COPD, chronic obstructive pulmonary disease.

**Fig. 4:**
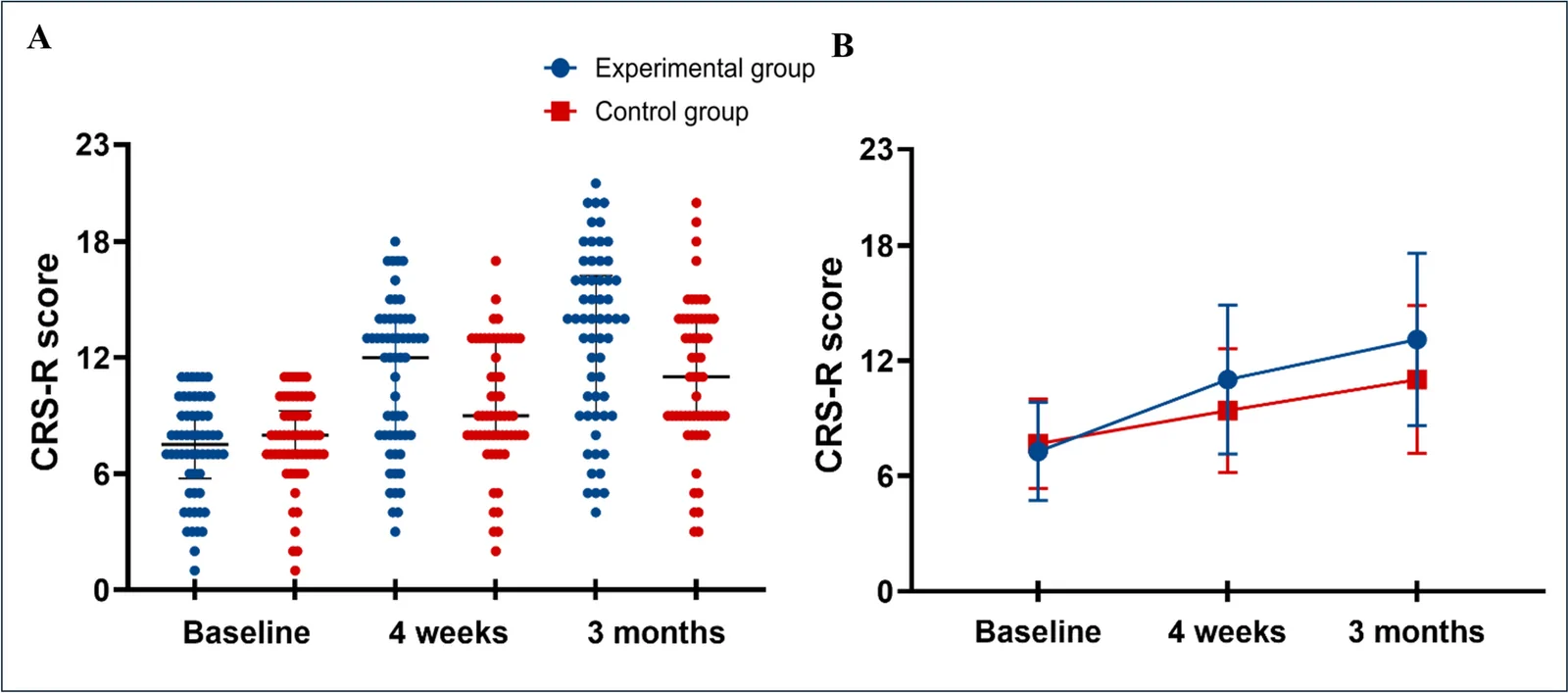
Median and interquartile range scatter plots (**A**) and mean ± standard deviation line charts (**B**) of CRS-R scores in both groups at 4 weeks postintervention and 3 months post-ABI

After conducting the Shapiro–Wilk test for normality, the CRS-R scores demonstrated a non-normal distribution (*P* < 0.05). In accordance with statistical methodology requirements, the CRS-R results in this study are primarily presented as median [IQR], with group comparisons performed using the Mann–Whitney *U* test. Additionally, mean ± standard deviation (Mean ± SD) is supplemented to provide information on the overall data distribution. At baseline, both groups demonstrated a comparable severity of consciousness impairment according to CRS-R scores (experimental group median: 7.5 [IQR: 5.8–9.0], mean ± SD: 7.28 ± 2.56; control group median: 8.0 [IQR: 7.0–9.3], mean ± SD: 7.67 ± 2.32; *P* = 0.462). Following the 4-week intervention, the experimental group exhibited a significantly greater increase in CRS-R scores (median: 12 [8–11], mean ± SD: 11.02 ± 3.87) compared with the control group (median: 9 [8–13], mean ± SD: 9.40 ± 3.23; *P* = 0.022). This significant difference persisted at the 3-month follow-up after ABI (experimental group median: 14 [9–16.3], mean ± SD: 13.10 ± 4.48; control group median: 11 [9–14], mean ± SD: 11.02 ± 3.85;* P* = 0.006) (Fig. 4).

**Fig. 5:**
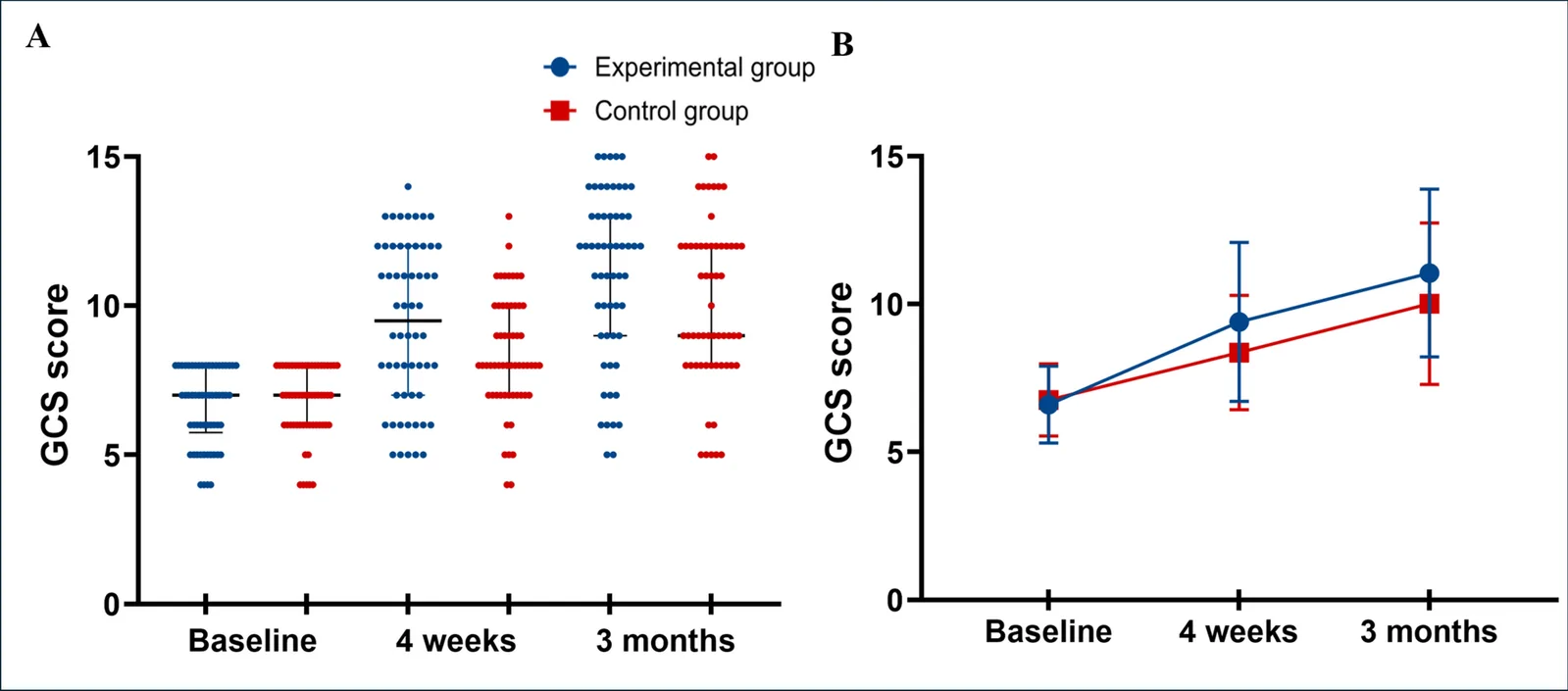
Median and interquartile range scatter plots (**A**) and mean ± standard deviation line charts (**B**) of GCS scores in both groups at 4 weeks postintervention and 3 months post-ABI

After conducting the Shapiro–Wilk test for normality, the GCS scores demonstrated a non-normal distribution (*P* < 0.05). In accordance with statistical methodology requirements, the GCS results in this study are primarily presented as median [IQR], with group comparisons performed using the Mann–Whitney *U* test. Additionally, mean ± standard deviation (Mean ± SD) is supplemented to provide information on the overall data distribution. In line with the main finding, similar baseline profiles in GCS scores at enrollment were observed in the two groups (*P* = 0.550). The experimental group had a median of 7.0 [IQR: 5.0–8.0] and a mean of 6.60 ± 1.30, whereas the control group had a median of 7.0 [IQR: 6.0–8.0] and a mean of 6.76 ± 1.22. Following 4 weeks of treatment, the experimental group showed a more pronounced increase in GCS scores with a median of 9.5 [IQR: 7–12] and a mean of 9.40 ± 2.69, which was significantly higher than the control group (median: 8 [IQR: 7–10]) and (mean: 8.36 ± 1.94;* P* = 0.029). This treatment effect was maintained during the follow-up period. During the 3-month evaluation, the experimental group had an overall median of 12 [IQR: 9–13] and a mean of 11.03 ± 2.87, which remains significantly higher than control values (median: 9 [IQR: 8–12], mean: 10.02 ± 2.73; *P* = 0.035), as shown in Fig. 5.

**Fig. 6:**
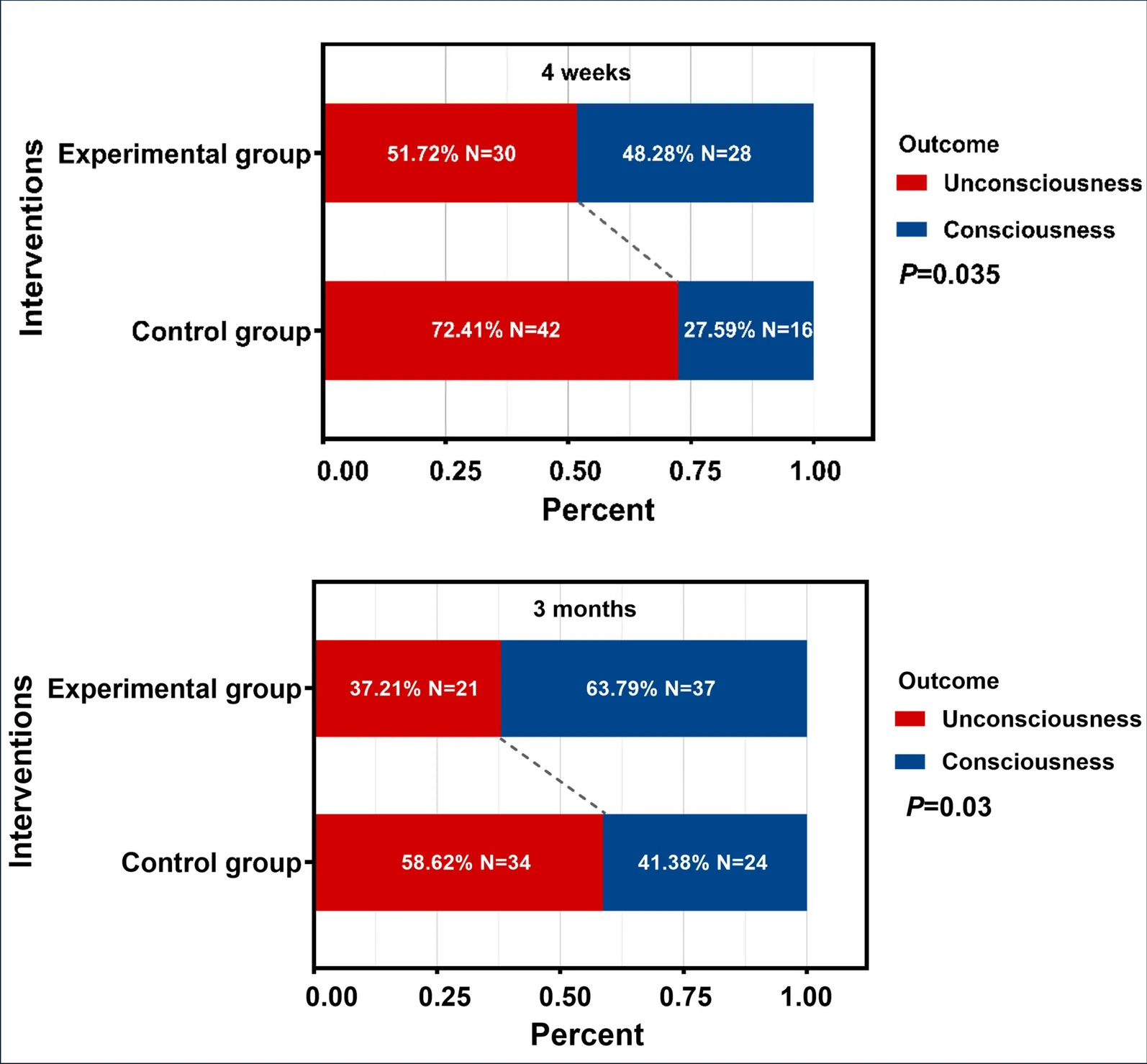
Percentage of patients who regained consciousness (MCS or higher) versus those who remained in VS/UWS or coma in both groups at 4 weeks postintervention and 3 months post-ABI

A significantly higher proportion of patients in the experimental group had regained consciousness by the 4-week assessment (*n* = 28, 48.28%; 95% CI 35.93–60.84%) compared with the control group (*n* = 16, 27.59%; 95% CI 17.75–40.21%), with an absolute difference of 20.69% (*P* = 0.035). This beneficial trend continued, with awakening rates further increasing at the 3-month follow-up. Ultimately, a significantly greater number of patients in the experimental group recovered consciousness (*n* = 37, 63.79%; 95% CI 50.93–74.95%) compared to the control group (*n* = 24, 41.38%; 95% CI 29.63–54.20%), and the between-group difference remained statistically significant *(P* = 0.03; Fig. 6).

**Fig. 7:**
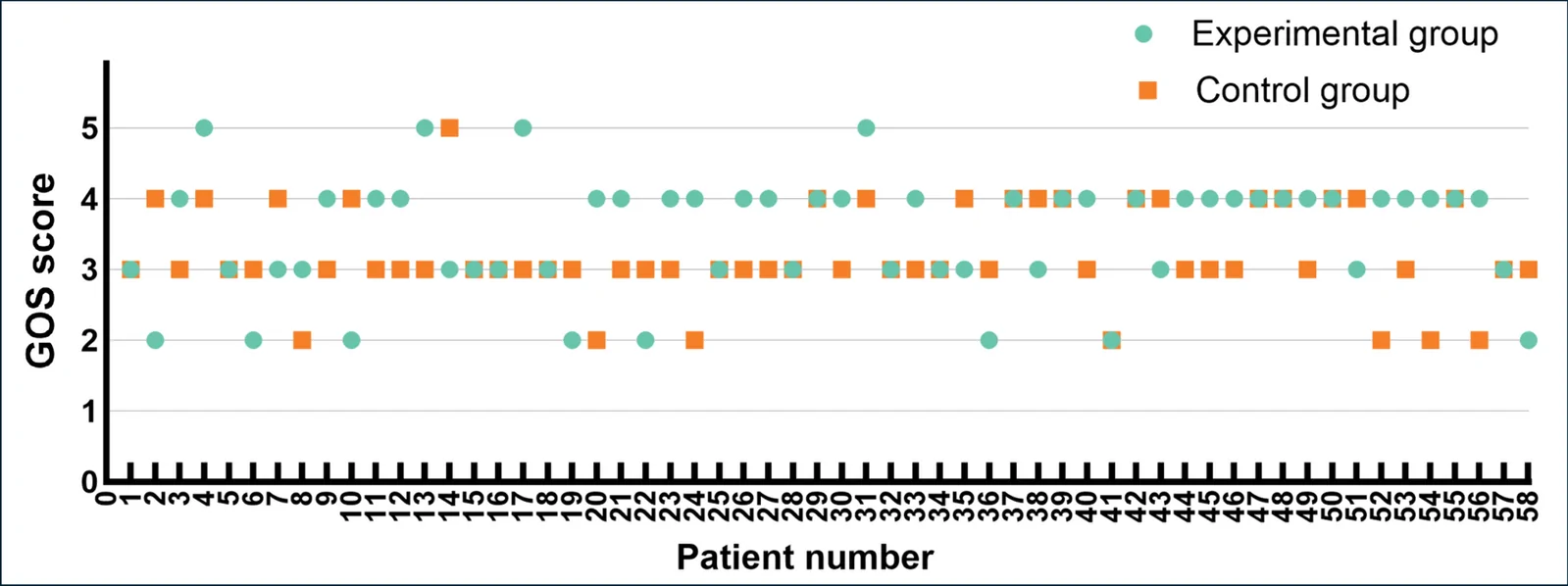
Distribution of individual GOS scores at 3 months post-ABI for the experimental (green circles) and control (orange squares) groups. Each data point represents one patient

Regarding long-term functional outcomes, the combined intervention likewise demonstrated a significant advantage. At the 3-month post-ABI follow-up, as shown in Fig. 7, the rate of positive outcome (GOS scores 4–5) was considerably higher in the patients receiving the experimental regimen (56.90% [33/58] vs. 31.03% [18/58]; absolute risk difference, 25.87; 95% CI 10.09–41.65; *P* = 0.009). In addition, the distribution of the overall GOS scores was skewed toward the more positive results in the experimental condition (median: 4 [IQR: 3–4]) compared with the control condition (median: 3 [IQR: 3–4]) that achieved statistical significance (Mann–Whitney *U* test, *P* = 0.023).

**Fig. 8:**
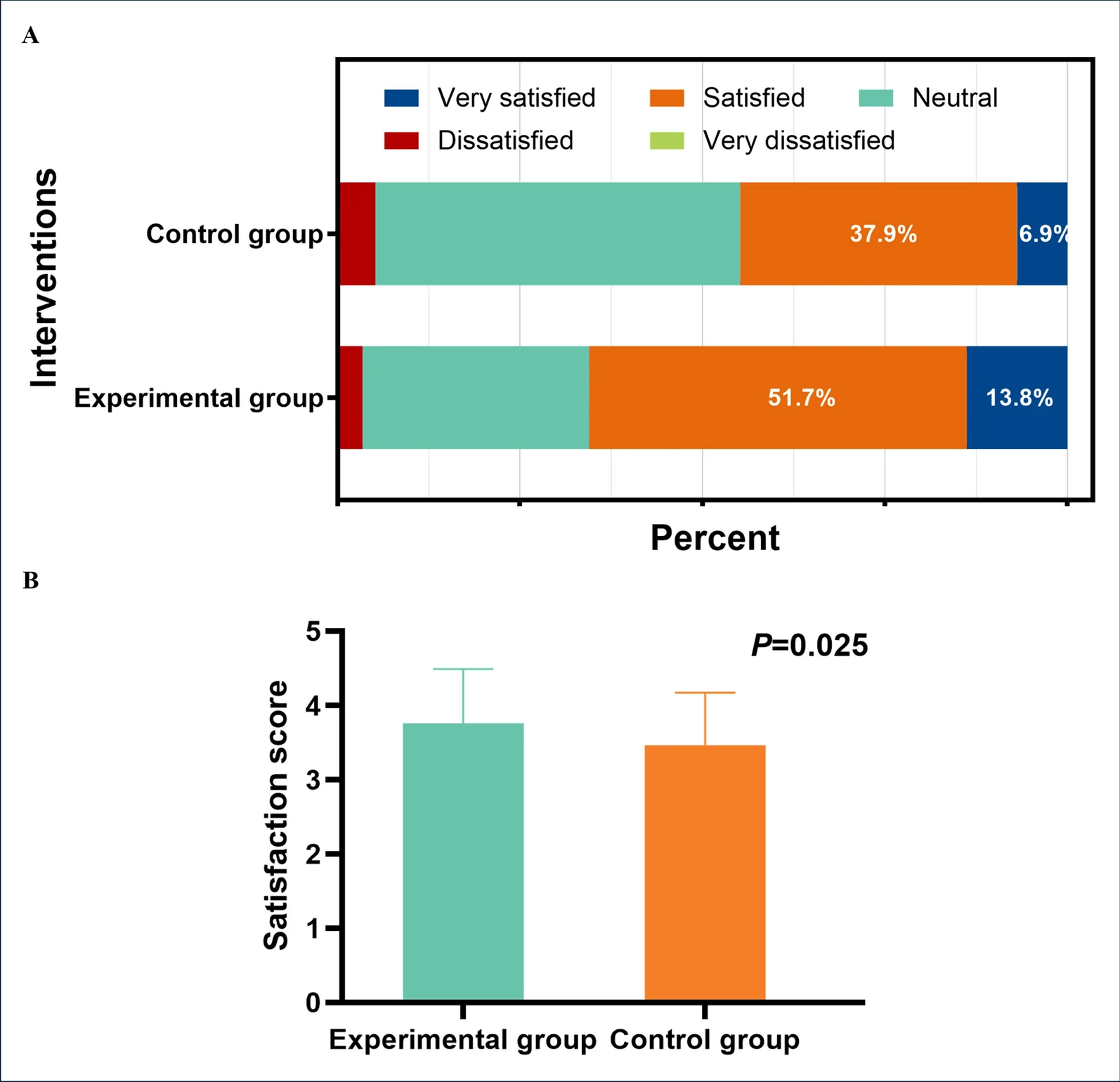
Assessment of family satisfaction at the 3-month follow-up. **A** Distribution of responses on a 5-point Likert scale (1: very dissatisfied to 5: very satisfied). **B** Intergroup comparison of mean satisfaction scores

The integrated intervention model gained greater approval among family members of patients. Family satisfaction was assessed at the 3-month follow-up and was defined as the caregiver’s subjective evaluation of the perceived therapeutic effectiveness of the intervention. According to the 5-point Likert scale test, the level of satisfaction was considerably higher in the experimental group than in the control group (Mann–Whitney *U* test, *P* = 0.025). The experimental group had a median satisfaction of 4 (IQR: 3–4), which was higher than the median of 3 (IQR: 3–4) in controls. Besides, 65.5% of the family members in the experimental group reported being satisfied (score 4) or very satisfied (score 5), a significantly higher percentage than that of the control group (44.8%; Fig. 8).

All patients were tolerant of the combined intervention regimen during the treatment period. No serious treatment-related adverse event was reported with any of the devices utilized in the study (disposable sterile acupuncture needles, MNES electrodes). There were no systemic risks such as seizures, arrhythmias, or elevated intracranial pressure associated with MNES, were observed. In the case of auricular acupuncture, there were no signs of allergies or local infections. In two patients, slight, nonpersistent erythema could be observed at the sites of electrode attachments, which subsided almost immediately after repositioning the electrodes, without causing any interruption in the treatment.

Nonintervention-related adverse events, including infections (pneumonia, urinary tract infections), fever, electrolyte imbalances, hypertension, and gastrointestinal issues, which are typical in this patient population, occurred in 12 patients (20.69%) in the experimental group and 10 patients (17.24%) in the control group. All were mild-to-moderate (Grade 1–2) and resolved with standard clinical management.

Fisher’s exact test showed no significant differences between groups in the overall AE rate (experimental: 14/58 [24.14%] vs. control: 10/58 [17.24%], *P* = 0.49), intervention-related AE rate (2/58 [3.45%] vs. 0/58 [0%], *P* = 0.50), or nonintervention-related AE rate (12/58 [20.69%] vs. 10/58 [17.24%], *P* = 0.81), confirming that the combined intervention did not increase the risk of adverse events.

During the study period, treatment interruptions owing to medical complications occurred in five patients (8.62%) in the experimental group (three with fever > 38.5 °C due to pulmonary infection, one with transient hypertension requiring medication adjustment, and one undergoing secondary surgery) and in three patients (5.17%) in the control group (two with fever > 38.5 °C due to pulmonary infection, one with transient hyperglycemia requiring insulin adjustment). Fisher’s exact test showed no significant difference in the incidence of interruptions between groups (*P* = 0.72), indicating that interruptions were balanced and unlikely to bias the treatment comparison. All interrupted patients resumed and completed the full treatment protocol after medical stabilization. Overall, the combined treatment regimen used in this study demonstrated good safety.

## Discussion

The present study aimed to investigate a consciousness-promoting intervention for patients with DOC after severe neurological diseases with an emphasis on the clinical needs and disease characteristics in the Gannan region of China. A high rate of DOC following ABI in Gannan translates into significant local hospital and societal burden. Moreover, TBI has been identified as a major risk factor of neurodegenerative disorders, such as dementia [39, 40]. Consequently, the creation of a successful treatment plan will be essential in enhancing the neurological outcome of patients with DOC.

CRS-R was chosen as the main end point in this study, which would be consolidated with the secondary measures of GCS scores, consciousness recovery rate, GOS scores, and family satisfaction to provide a multi-dimensional analysis of intervention impact. The CRS-R has been dubbed the gold standard of behavioral measurement of DOC worldwide owing to its unmatched sensitivity in detecting subtle and changing signs of awareness [41]. This accuracy is essential to the correct identification of the MCS and VS/UWS, which is a core diagnostic issue in this patient population. It is the internationally prescribed device for this purpose. The GCS has limitations on separating multiple levels of consciousness, despite its widespread use in acute assessment [42], and this is why it was included as a secondary end point in order to balance sensitivity and clinical applicability.

The percentage of patients who regained consciousness, a secondary end point, is a clinically important shift in patient status. It is an objective and clinically significant measure of the ability of treatment to meaningfully affect the level of consciousness. The GOS score is a widely used measure of long-term functional outcome, and early GOS assessment (e.g., at 3 months postinjury) has been shown to predict long-term neurologic functional independence [43, 44]. The concept of family satisfaction describes how caregivers view treatment and the humanitarian care perspective. The recovery process of patients with DOC is quite complex and cannot be fully captured by a single evaluation index. This research utilized a multidimensional and multi-time point assessment system [45, 46], which has been recommended in international neurological rehabilitation guidelines, assuring scientific rigor, clinical relevance and fulfilling objective evaluation demands of the contemporary bio-psycho-social medical model.

The research revealed that the MNES combined with the auricular intradermal needle treatment was associated with significantly greater improvements in CRS-R scores, GOS scores, and percentages of patients who recovered consciousness both at 4 weeks and at 3 months across various time points, compared with the single MNES treatment group. The current findings have preliminary clinical implications in the treatment of DOC, underscoring the preliminary clinical advantages of this integrated modality in promoting functional neurological recovery, which warrant further validation in larger-scale, multi-center controlled trials.

Consciousness restoration is a critical milestone in DOC management, but it represents only one component of the multifaceted recovery process after severe brain injury. In patients with widespread structural brain damage, functional outcomes following consciousness recovery are highly variable. Our findings suggest that the combined intervention not only promoted consciousness recovery but also improved functional outcomes, as reflected by significantly higher GOS scores. This may be attributed to the intervention’s ability to create a more favorable neural environment, enabling subsequent neuroplasticity and functional rehabilitation. Future research with longer follow-up is warranted to further elucidate the relationship between consciousness recovery and long-term functional independence.

As reported in previous studies, the effectiveness of MNES in DOC is explained by its multi-level mechanisms. First, electrical activation of the median nerve triggers a cascade of neural events, starting with depolarization that progresses to activation of the ARAS and extensive cortical arousal. The modulated release of important neuromodulators, namely acetylcholine, norepinephrine, and dopamine, facilitates this process, as they combine to increase neural excitability and reestablish conscious awareness [3, 47]. In addition to these electrophysiological outcomes, MNES facilitates cerebral blood flow and oxygen delivery, which help to restore vulnerable neurons within the ischemic penumbra. Molecular evidence also indicates that its advantages can include the stimulation of brain-derived neurotrophic factor (BDNF) and inhibition of neuronal ferroptosis, two pathways that are known to play a critical role in cell survival and repair [48]. MNES is reportedly well-tolerated and large-scale studies have not proven a significant association with severe adverse events such as seizures or arrhythmias, further highlighting its safety superiority over many pharmacological or invasive interventions [4]. Moreover, MNES is easy to conduct, inexpensive, and can be implemented and popularized with ease in intensive care units and general wards.

Auricular acupuncture plays an important role in the history of TCM. According to the Huangdi Neijing, the ear is a meeting point of meridian Qi, and the stimulation of the auricular points can bring the meridians into harmony and positively influence the circulation of Qi and blood all over the body. Among the described benefits of needle-embedding acupuncture therapy in the Suwen are the ability to retain longer and to constantly stimulate without pressing. This allows less workload on the caregiver and is particularly helpful with patients who have low self-care skills.

In modern medical terms, the area around the ear is richly supplied and is innervated by branches of the vagus and trigeminal nerves. Based on previous mechanistic studies, auricular stimulation has been suggested to influence recovery of impaired consciousness through modulation of neurophysiological pathways linked to the auricular branch of the vagus nerve (ABVN). Auricular stimulation activates afferent projections of the ABVN to the nucleus tractus solitarius (NTS) and associated brainstem nuclei, which are connected with ascending arousal systems and large-scale cortical networks implicated in conscious processing [49, 50]. These peripheral nerves create widespread neurovascular networks with the central nervous system and are believed to regulate various neurotransmitters, such as serotonin and β-endorphins, and effect activities within cognitive brain networks [51–53] In addition to its key effects, functional near-infrared spectroscopy (fNIRS) experiments have shown that functional brain activity changes can be recorded in response to auricular stimulation, especially in the default mode network and prefrontal cortex [32]. While these findings suggest that auricular stimulation could influence higher-order brain networks, it is important to note that these mechanistic insights are drawn from the literature and were not directly tested in the present study, which lacked neurophysiological measures such as electroencephalography (EEG), functional magnetic resonance imaging (fMRI), or autonomic assessments.

In comparison with traditional forms of acupuncture, such as the use of acupuncture points on the ear, needle-embedding acupuncture offers amazing benefits, including sustained stimulation without much intervention. This helps lessen the workload of caregivers and makes it very practical when being used in the primary healthcare context [54].

Enhanced effects of MNES and auricular intradermal needle were observed, with the two modalities complementing each other in mechanisms, stimulation regimes, and persistence time. MNES offers a strong stimulus in the short term, quickly eliciting a central nervous response, and auricular acupuncture offers ongoing stimulation, which has to balance long-term treatment duration/short-stimulation time of MNES. Combining these two regimens results in complementary effects that help to maintain brain plasticity and facilitate long-term neurological recovery by promoting both consciousness restoration and creating conditions conducive to subsequent functional improvement. Auricular needling is essentially painless and has low infection risk because of its aseptic procedure, and MNES does not raise the incidence of significant adverse events, including seizures or arrhythmias. In the study, a small number of patients had mild and transient erythema at electrode attachment sites, which corrected with replacement of electrodes, and did not result in treatment discontinuation. This is a safe, cost-effective, long-term treatment, and the combined treatment regimen is promising for clinical use.

## Limitations

Despite the favorable outcomes of this research in examining the application of DOC, some limitations exist. First, our study lacked a sham auricular stimulation or attention-control arm, which prevents definitive differentiation between the specific neuromodulatory effects of auricular intradermal needle stimulation and nonspecific contributing factors such as increased caregiver interaction or placebo-related responses. As such, the observed additive benefit of combining auricular needling with MNES cannot be exclusively attributed to the specific neural effects of auricular stimulation. Future randomized controlled trials should incorporate sham-controlled or auricular-only designs to clarify the specific therapeutic contribution of auricular stimulation. The study included patients with GCS scores ranging from 4 to 8 at enrollment, which encompasses a spectrum of disorders of consciousness including coma, VS/UWS, and potentially MCS. Therefore, the generalizability of our findings to patients with milder impairment or higher GCS scores requires further investigation. Additionally, the relatively small sample size (*n* = 116) precluded robust subgroup analyses based on etiology of brain injury. Future studies with larger cohorts are needed to explore whether treatment response differs between traumatic and nontraumatic etiologies. Also, the follow-up was restricted to 4 weeks and 3 months, which might not be sufficient to capture the longitudinal nature of recovery. Because recovery is generally a rather slow process, long-term observation (6 months to 1 year) would give us a good understanding of the long-term effectiveness of this practice. Moreover, the differentiation of treatment procedures could have affected the outcomes, thus prospective research should tackle these constraints to maximize the output of treatment. Additionally, while our study demonstrated statistically significant improvements in CRS-R scores in the combined intervention group, we did not assess whether these changes exceed established thresholds for minimal clinically important difference or minimal detectable change as recently proposed for the CRS-R [55, 56]. Future studies should incorporate these indices to better interpret the clinical meaningfulness of observed treatment effects.

Furthermore, while auricular intradermal needle stimulation is rooted in TCM, its anatomical basis, innervation of the auricular region by the vagus and trigeminal nerves, is universal. The procedure is relatively simple and could be implemented in diverse clinical settings following standardized training programs and protocol development. Future research should focus on developing standardized training curricula and point selection protocols to facilitate wider adoption of this intervention beyond TCM practice.

## Conclusions

This study is one of the preliminary exploratory studies to evaluate the efficacy of combined MNES and auricular intradermal needle in patients with DOC following ABI. The results demonstrated that the combined treatment significantly outperformed single MNES treatment in CRS-R scores, GCS scores, percentage of patients who regained consciousness, GOS scores, and family satisfaction, suggesting that multimodal sensory stimulation has complementary effects. This study provides a preliminary, innovative, and feasible exploratory strategy for consciousness restoration therapy in patients with DOC, which warrants further validation in large-scale, multi-center, and controlled trials.

## Supplementary Information

Below is the link to the electronic supplementary material.

Supplementary file1 (DOCX 40 KB)

Supplementary file2 (XLSX 27 KB)

## Data Availability

Data is provided within the manuscript or supplementary information files.

## References

[1] Bai Y, Lin Y, Ziemann U. Managing disorders of consciousness: the role of electroencephalography. J Neurol. 2021;268(11):4033–65. 10.1007/s00415-020-10095-z. PMC8505374 32915309

[2] Edlow BL, Claassen J, Schiff ND, Greer DM. Recovery from disorders of consciousness: mechanisms, prognosis and emerging therapies. Nat Rev Neurol. 2021;17(3):135–56. 10.1038/s41582-020-00428-x. PMC7734616 33318675

[3] Liu YS, Liu ZB, Yang Z, Zhao L, Li HL. Clinical efficacy of hyperbaric oxygen combined with different timings of right median-nerve electrical stimulation in patients with brain injury-induced disorders of consciousness. Brain Behav. 2022;12(9):e2716. 10.1002/brb3.2716. PMC9480931 35920129

[4] Gu C, Shang H, Kong Y, Peng M, Jiang H, Wang S, et al. Safety of median nerve electrical stimulation in disorders of consciousness: a systematic review and meta-analysis of randomized controlled trials. PLoS One. 2025;20(7):e0324046. 10.1371/journal.pone.0324046. PMC12312889 40743130

[5] Johnson WD, Griswold DP. Traumatic brain injury: a global challenge. Lancet Neurol. 2017;16(12):949–50. 10.1016/S1474-4422(17)30362-9. 29122521

[6] Karagianni MD, Brotis AG, Gatos C, Kalamatianos T, Vrettou C, Stranjalis G, et al. Neuromonitoring in severe traumatic brain injury: a bibliometric analysis. Neurocrit Care. 2022;36(3):1044–52. 10.1007/s12028-021-01428-5. 35075580

[7] Maas AIR, Menon DK, Adelson PD, Andelic N, Bell MJ, Belli A, et al. Traumatic brain injury: integrated approaches to improve prevention, clinical care, and research. Lancet Neurol. 2017;16(12):987–1048. 10.1016/S1474-4422(17)30371-X. 29122524

[8] Cain JA, Spivak NM, Coetzee JP, Crone JS, Johnson MA, Lutkenhoff ES, et al. Ultrasonic deep brain neuromodulation in acute disorders of consciousness: a proof-of-concept. Brain Sci. 2022;12(4):428. 10.3390/brainsci12040428. PMC9032970 35447960

[9] Thibaut A, Schiff N, Giacino J, Laureys S, Gosseries O. Therapeutic interventions in patients with prolonged disorders of consciousness. Lancet Neurol. 2019;18(6):600–14. 10.1016/S1474-4422(19)30031-6. 31003899

[10] Raguz M, Predrijevac N, Dlaka D, Oreskovic D, Rotim A, Romic D, et al. Structural changes in brains of patients with disorders of consciousness treated with deep brain stimulation. Sci Rep. 2021;11(1):4401. 10.1038/s41598-021-83873-y. PMC7902623 33623134

[11] Giacino JT, Whyte J, Bagiella E, Kalmar K, Childs N, Khademi A, et al. Placebo-controlled trial of amantadine for severe traumatic brain injury. N Engl J Med. 2012;366(9):819–26. 10.1056/NEJMoa1102609. 22375973

[12] Giacino JT, Katz DI, Schiff ND, Whyte J, Ashman EJ, Ashwal S, et al. Practice guideline update recommendations summary: disorders of consciousness: report of the Guideline Development, Dissemination, and Implementation Subcommittee of the American Academy of Neurology; the American Congress of Rehabilitation Medicine; and the National Institute on Disability, Independent Living, and Rehabilitation Research. Neurology. 2018;91(10):450–60. 10.1212/WNL.0000000000005926. PMC6139814 30089618

[13] Wan X, Zhang Y, Li Y, Song W. An update on noninvasive neuromodulation in the treatment of patients with prolonged disorders of consciousness. CNS Neurosci Ther. 2024;30(5):e14757. 10.1111/cns.14757. PMC11094579 38747078

[14] Murtaugh B, Morrissey AM, Fager S, Knight HE, Rushing J, Weaver J. Music, occupational, physical, and speech therapy interventions for patients in disorders of consciousness: an umbrella review. NeuroRehabilitation. 2024;54(1):109–27. 10.3233/NRE-230149. 38277314

[15] Wu X, Xie L, Lei J, Yao J, Li J, Ruan L, et al. Acute traumatic coma awakening by right median nerve electrical stimulation: a randomised controlled trial. Intensive Care Med. 2023;49(6):633–44. 10.1007/s00134-023-07072-1. PMC10182548 37178149

[16] Zhou Y, Yang H, You M, Feng Z, Dong X. Cognition-enhancement effect of median nerve electrical stimulation in patients with cognitive impairment: a retrospective cohort study. World Neurosurg. 2024;184:e537–45. 10.1016/j.wneu.2024.01.165. 38320650

[17] Gottshall JL, Rossi Sebastiano D. Sleep in disorders of consciousness: diagnostic, prognostic, and therapeutic considerations. Curr Opin Neurol. 2020;33(6):684–90. 10.1097/WCO.0000000000000870. 33177374

[18] An L, Shao R, Wang X, Tang Z. Research progress on median nerve electrical stimulation for awakening comatose patients with brain injury. Zhonghua Wei Zhong Bing Ji Jiu Yi Xue Za Zhi. 2024;36(9):997–1000. 10.3760/cma.j.cn121430-20240617-00513. 39380525

[19] Wang P, Cao W, Zhou H, Zhang HX, Zhang L, Liu L, et al. Efficacy of median nerve electrical stimulation on the recovery of patients with consciousness disorders: a systematic review and meta-analysis. J Int Med Res. 2022;50(11):3000605221134467. 10.1177/03000605221134467. PMC9720824 36448965

[20] Huo C, Li X, Jing J, Ma Y, Li W, Wang Y, et al. Median nerve electrical stimulation-induced changes in effective connectivity in patients with stroke as assessed with functional near-infrared spectroscopy. Neurorehabil Neural Repair. 2019;33(12):1008–17. 10.1177/1545968319875952. 31550986

[21] Yang J, Li X, Yang X, Zhu T, Ou S. Acute traumatic coma awakening induced by median nerve electrical stimulation: a systematic review and meta-analysis. Neurocrit Care. 2025;42(3):817–28. 10.1007/s12028-024-02141-9. 39448428

[22] Yang T, Wang S, Zhang X, Liu L, Liu Y, Zhang C. Efficacy of auricular acupressure in maintenance haemodialysis patients: a systematic review and meta-analysis. J Clin Nurs. 2022;31(5–6):508–19. 10.1111/jocn.15966. 34268817

[23] Guo K, Lu Y, Wang X, Duan Y, Li H, Gao F, et al. Multi-level exploration of auricular acupuncture: from traditional Chinese medicine theory to modern medical application. Front Neurosci. 2024;18:1426618. 10.3389/fnins.2024.1426618. PMC11456840 39376538

[24] Kong X, Fang H, Li X, Zhang Y, Guo Y. Effects of auricular acupressure on dysmenorrhea: a systematic review and meta-analysis of randomized controlled trials. Front Endocrinol (Lausanne). 2022;13:1016222. 10.3389/fendo.2022.1016222. PMC9851274 36686444

[25] Mao JJ, Liou KT, Baser RE, Bao T, Panageas KS, Romero SAD, et al. Effectiveness of electroacupuncture or auricular acupuncture vs usual care for chronic musculoskeletal pain among cancer survivors: the PEACE randomized clinical trial. JAMA Oncol. 2021;7(5):720–7. 10.1001/jamaoncol.2021.0310. PMC7974834 33734288

[26] Ozum U, Akyol M, Balaban H, Ozdemir E, Soydan S. Effect of cervical spinal cord electrical stimulation on nitric oxide levels in brain and dermal tissues: an evaluation using by real-time nitric oxide measurement. Acta Neurochir (Wien). 2012;154(9):1641–6. 10.1007/s00701-012-1331-3. 22454038

[27] Edlow BL, Sanz LRD, Polizzotto L, Pouratian N, Rolston JD, Snider SB, et al. Therapies to restore consciousness in patients with severe brain injuries: a gap analysis and future directions. Neurocrit Care. 2021;35(Suppl 1):68–85. 10.1007/s12028-021-01227-y. PMC8266715 34236624

[28] Benghanem S, Mazeraud A, Azabou E, Chhor V, Shinotsuka CR, Claassen J, et al. Brainstem dysfunction in critically ill patients. Crit Care. 2020;24(1):5. 10.1186/s13054-019-2718-9. PMC6945639 31907011

[29] Cooper EB, Scherder EJ, Cooper JB. Electrical treatment of reduced consciousness: experience with coma and Alzheimer’s disease. Neuropsychol Rehabil. 2005;15(3–4):389–405. 10.1080/09602010443000317. 16350980

[30] Liu JT, Lee JK, Chang PJ, Sun CM. Increased regional cerebral perfusion in contralateral motor and somatosensory areas after median nerve stimulation therapy. Acta Neurochir Suppl. 2008;101:65–70. 10.1007/978-3-211-78205-7_11. 18642636

[31] Zhong Q, Wang D, Bai YM, Du SZ, Song YL, Zhu J. Effectiveness of auricular acupressure for acute postoperative pain after surgery: a systematic review and meta-analysis. Chin J Integr Med. 2019;25(3):225–32. 10.1007/s11655-019-3063-1. 30815805

[32] Zhang J, Huang W, Chen Z, Jiang H, Su M, Wang C. Effect of auricular acupuncture on neuroplasticity of stroke patients with motor dysfunction: a fNIRS study. Neurosci Lett. 2023;812:137398. 10.1016/j.neulet.2023.137398. 37468089

[33] Gao G. Scans: a story of Shanghai coma afferent nerve stimulation on traumatic coma. In: Journal of Neurotrauma: 2022: Mary Ann Liebert, Inc; 2022

[34] American Congress of Rehabilitation Medicine BI-ISIGDoCTF, Seel RT, Sherer M, Whyte J, Katz DI, Giacino JT, et al. Assessment scales for disorders of consciousness: evidence-based recommendations for clinical practice and research. Arch Phys Med Rehabil. 2010;91(12):1795–813. 10.1016/j.apmr.2010.07.218. 21112421

[35] Bodien YG, Carlowicz CA, Chatelle C, Giacino JT. Sensitivity and specificity of the coma recovery scale—Revised total score in detection of conscious awareness. Arch Phys Med Rehabil. 2016;97(3):490-492 e491. 10.1016/j.apmr.2015.08.422. PMC5018674 26342571

[36] Kebapci A, Dikec G, Topcu S. Interobserver reliability of Glasgow Coma Scale Scores for intensive care unit patients. Crit Care Nurse. 2020;40(4):e18–26. 10.4037/ccn2020200. 32737493

[37] Veldeman M, Rossmann T, Haeren R, Vossen LV, Weiss M, Conzen C, et al. Delayed cerebral infarction after aneurysmal subarachnoid hemorrhage: location, distribution patterns, infarct load, and effect on outcome. Neurology. 2024;103(3):e209607. 10.1212/WNL.0000000000209607. 38950352

[38] Ma J, Huang X, Hu Y, Xu B, Jin C. Clinical value of serum secretoneurin levels in prediction of delayed cerebral ischemia and prognostic analysis of aneurysmal subarachnoid hemorrhage: a prospective cohort study. Int J Gen Med. 2024;17:3555–73. 10.2147/IJGM.S469287. PMC11334926 39165486

[39] Graham NS, Sharp DJ. Dementia after traumatic brain injury. BMJ. 2023;383:2065. 10.1136/bmj.p2065. 37857435

[40] Livingston G, Huntley J, Sommerlad A, Ames D, Ballard C, Banerjee S, et al. Dementia prevention, intervention, and care: 2020 report of the Lancet Commission. Lancet. 2020;396(10248):413–46. 10.1016/S0140-6736(20)30367-6. PMC7392084 32738937

[41] Magnani FG, Barbadoro F, Cacciatore M, Leonardi M. The importance of instrumental assessment in disorders of consciousness: a comparison between American, European, and UK international recommendations. Crit Care. 2022;26(1):245. 10.1186/s13054-022-04119-5. PMC9367125 35948933

[42] Bodien YG, Barra A, Temkin NR, Barber J, Foreman B, Vassar M, et al. Diagnosing level of consciousness: the limits of the Glasgow Coma Scale total score. J Neurotrauma. 2021;38(23):3295–305. 10.1089/neu.2021.0199. PMC8917895 34605668

[43] Miller KJ, Schwab KA, Warden DL. Predictive value of an early Glasgow outcome scale score: 15-month score changes. J Neurosurg. 2005;103(2):239–45. 10.3171/jns2005.103.2.0239. 16175852

[44] King JT Jr., Carlier PM, Marion DW. Early Glasgow outcome scale scores predict long-term functional outcome in patients with severe traumatic brain injury. J Neurotrauma. 2005;22(9):947–54. 10.1089/neu.2005.22.947. 16156710

[45] Giacino JTKD, Schiff ND, Whyte J, Ashman EJ, Ashwal S, Barbano R, et al. Practice guideline update recommendations summary: disorders of consciousness: report of the Guideline Development, Dissemination, and Implementation Subcommittee of the American Academy of Neurology; the American Congress of Rehabilitation Medicine; and the National Institute on Disability, Independent Living, and Rehabilitation Research. Neurology. 2019;93(3):135. 10.1212/WNL.0000000000007382. PMC7673332 31308171

[46] Noe E, Navarro MD, Moliner B, O’Valle M, Olaya J, Maza A, et al. Guideline: neurorehabilitation in patients with disorder of consciousness. Recommendations from the Spanish Society of Neurorehabilitation. Neurologia (Engl Ed). 2025;40(1):92–117. 10.1016/j.nrleng.2025.01.002. 39800160

[47] Zhao K, Liu A. Effect of right median nerve stimulation on clinical efficacy and prognosis of patients with consciousness disorder caused by traumatic brain injury. J Fujian Univ Tradit Chin Med. 2020;30(03):192–6.

[48] Zhong YJ, Liu LL, Zhao Y, Feng Z, Liu Y. Elucidating the molecular mechanisms behind the therapeutic impact of median nerve stimulation on cognitive dysfunction post-traumatic brain injury. Exp Gerontol. 2024;194:112500. 10.1016/j.exger.2024.112500. 38901771

[49] He W, Wang X, Shi H, Shang H, Li L, Jing X, et al. Auricular acupuncture and vagal regulation. Evid Based Complement Alternat Med. 2012;2012:786839. 10.1155/2012/786839. PMC3523683 23304215

[50] Briand MM, Gosseries O, Staumont B, Laureys S, Thibaut A. Transcutaneous auricular vagal nerve stimulation and disorders of consciousness: a hypothesis for mechanisms of action. Front Neurol. 2020;11:933. 10.3389/fneur.2020.00933. PMC7477388 32982941

[51] Wang Y, Li SY, Wang D, Wu MZ, He JK, Zhang JL, et al. Transcutaneous auricular vagus nerve stimulation: from concept to application. Neurosci Bull. 2021;37(6):853–62. 10.1007/s12264-020-00619-y. PMC8192665 33355897

[52] Li Y, Du JL, Hao PL, Zhang MX, Jiang YB, Shu M, et al. Effect of auricular point sticking therapy on perioperative pain in patients with partial lung resection. Zhongguo Zhen Jiu. 2021;41(6):603–7. 10.13703/j.0255-2930.20200505-k0003. 34085475

[53] Wang H, Liu XR, Wu XJ, He TZ, Miao D, Jiang JF, et al. Additional value of auricular intradermal acupuncture alongside selective serotonin reuptake inhibitors: a single-blinded, randomized, sham-controlled preliminary clinical study. Acupunct Med. 2021;39(6):596–602. 10.1177/0964528421997155. 33657871

[54] Nielsen A, Gereau S, Tick H. Risks and safety of extended auricular therapy: a review of reviews and case reports of adverse events. Pain Med. 2020;21(6):1276–93. 10.1093/pm/pnz379 32430505

[55] Weaver JA, Cogan AM, Kozlowski AJ, Grady-Dominguez P, O’Brien KA, Bodien YG, et al. Interpreting change in disorders of consciousness using the Coma Recovery Scale-Revised. J Neurotrauma. 2024;41(15–16):e1996–2008. 10.1089/neu.2023.0567. PMC11386986 38613812

[56] Grady-Dominguez P, Bodien YG, O’Brien KA, Giacino JT, Weaver JA. Evaluating the hierarchy of rating scale categories for the Coma Recovery Scale-Revised in non-traumatic brain injury: a Rasch analysis. Neurorehabil Neural Repair. 2026;40(1):37–48. 10.1177/15459683251399142. 41451652

