# Supplementary material for: Promoting Consciousness Recovery in Patients with Acquired Brain Injury Through Multimodal Sensory Stimulation: A Randomized Controlled Trial of Median Nerve and Auricular Stimulation
Source: Neurocrit Care. 2026 May 28;45(2):833–49. doi: 10.1007/s12028-026-02543-x (PMC13642423; doi:10.1007/s12028-026-02543-x)
Supplement: Supplementary file 1 [file 12028_2026_2543_MOESM1_ESM.docx]

**Supplementary Material**

***for***

**NIR-Driven Fer-1@PB Nanoparticles for Treatment of Sepsis-Associated Acute Lung Injury by Adjusting**

**CXCL2/ARF6/NCF1-Mediated Immune Response**

Hai Lia #, Wei Song b #, Shuping Zeng c, Ling Liud , Changyu Liue, Hanjing Luf, g*

a Department of Rehabilitation Medicine, The First Affiliated Hospital of Gannan Medical University, Ganzhou 341000, China.

b Department of Rehabilitation Medicine, Shanghai Fourth People's Hospital, School of Medicine, Tongji University, Shanghai 200000, China.

c Department of Rehabilitation Medicine, The First People's Hospital of Nankang , Ganzhou City 341411, China.

d Department of Gastroenterology, The First School of Clinical Medicine, Gannan Medical University, Ganzhou 341000, China.

e Department of Anesthesiology, The First School of Clinical Medicine, Gannan Medical University, Ganzhou 341000, China.

f Department of Emergency Medicine, The First Affiliated Hospital of Gannan Medical University, Ganzhou 341000, China.

g Key Laboratory of Emergency and Trauma of Ministry of Education, Key Laboratory of Hainan Trauma and Disaster Rescue, Department of Emergency Medicine, The First Affiliated Hospital, Hainan Medical University ，Haikou 570102, China.

\# These authors contributed equally to this work.

*Corresponding author: Hanjing Lu.

Corresponding author: Department of Emergency Medicine, The First Affiliated Hospital of Gannan Medical University, Ganzhou 341000, China.

Key Laboratory of Emergency and Trauma of Ministry of Education, Key Laboratory of Hainan Trauma and Disaster Rescue, Department of Emergency Medicine, The First Affiliated Hospital, Hainan Medical University ，Haikou 570311, China.

E-mail addresses: 527447713@qq.com (H. Lu).

The First Affiliated Hospital of Gannan Medical University Department of Rehabilitation Medicine

**Modified Coma Recovery Scale (CRS-R)**

Name: ________ Gender: ____ Age: ____ Department & Bed Number: ________

Hospitalization Number: ________ Diagnosis:

| **Item** | **Examination Content** | **Score** | **First**  **Assessment** | **Second**  **Assessment** | **Third**  **Assessment** |
| --- | --- | --- | --- | --- | --- |
| Auditory | Stable response to commands ★ | 4 |  |  |  |
|  | Able to repeatedly  follow commands ★ | 3 |  |  |  |
|  | Sound localization: turning head/gazing | 2 |  |  |  |
|  | Blink response to  sound (startle response) | 1 |  |  |  |
|  | None | 0 |  |  |  |
| Visual | Object recognition ★ | 5 |  |  |  |
|  | Object localization:  reaching for objects ★ | 4 |  |  |  |
|  | Eye tracking ★ | 3 |  |  |  |
|  | Visual target  localization: gazing (＞ 2 seconds) ★ | 2 |  |  |  |
|  | Blink response to  threat (startle response) | 1 |  |  |  |

| **Item** | **Examination Content** | **Score** | **First**  **Assessment** | **Second**  **Assessment** | **Third**  **Assessment** |
| --- | --- | --- | --- | --- | --- |
|  | None | 0 |  |  |  |
| Motor | Functional object use • | 6 |  |  |  |
|  | Voluntary motor response ★ | 5 |  |  |  |
|  | Able to manipulate objects ★ | 4 |  |  |  |
|  | Pain localization ★ | 3 |  |  |  |
|  | Limb withdrawal due to pain | 2 |  |  |  |
|  | Abnormal posture  (hyperflexion/hyperext ension) due to pain | 1 |  |  |  |
|  | No response to painful stimuli | 0 |  |  |  |
| Verbal | Comprehensible verbal expression ★ | 3 |  |  |  |
|  | Vocalization/vocalizing movements | 2 |  |  |  |
|  | Reflexive vocalizing movements | 1 |  |  |  |
|  | None | 0 |  |  |  |

| **Item** | **Examination Content** | **Score** | **First**  **Assessment** | **Second**  **Assessment** | **Third**  **Assessment** |
| --- | --- | --- | --- | --- | --- |
| Communica  tion | Fully accurate  communication • | 2 |  |  |  |
|  | Incompletely accurate communication ★ | 1 |  |  |  |
|  | None | 0 |  |  |  |
| Arousal  Level | Able to pay attention | 3 |  |  |  |
|  | Able to open eyes | 2 |  |  |  |
|  | Eye opening with stimulation | 1 |  |  |  |
|  | None | 0 |  |  |  |
| Total Score | |  |  |  |  |
| Assessment Time | |  |  |  |  |
| Assessor | |  |  |  |  |

Note:★ Minimally Conscious State (MCS)

• Emergence from Minimally Conscious State (EMCS)

The First Affiliated Hospital of Gannan Medical University Department of Rehabilitation Medicine

**Glasgow Coma Scale (GCS)**

Name: Gender: Age: Department & Bed Number:

________	____	____	________

Hospitalization Number:

Diagnosis:

**Patient's Response**

**Score**

4

3

2

1

6

**Item**

Eye Opening (E)

**Test**

Spontaneous Verbal Stimuli

Pain

Pain

Obeying Commands

Opens eyes by oneself spontaneously

Opens eyes when the examiner asks

questions in a loud voice

Opens eyes when pinched (in response

to pain)

Does not open eyes when pinched (no

response to pain)

Able to perform simple commands

Motor Response

(M)

Shows a response of attempting to

escape the painful stimulus when

Pain

5

pinched

Abducts the shoulders when the nail bed

Pain

4

is pinched (in response to pain)

Presents with decorticate rigidity (Upper

limbs: flexion, adduction, internal

rotation; Lower limbs: extension,

Pain

3

adduction, internal rotation, ankle

plantar flexion) when pinched (in

response to pain)

Presents with decerebrate rigidity

(Upper limbs: extension, adduction,

internal rotation, wrist and finger

Pain

2

flexion; Lower limbs: same as

decorticate rigidity) when pinched (in

response to pain)

Shows no response at all when pinched

Pain

1

(no motor response to pain)

Able to converse correctly, and can tell

the examiner where he is, who he is, as

Verbal Response

(V)

Verbal Communication

5

well as the year and month (with

accurate orientation)

Able to speak, but with confused speech

Verbal Communication

Verbal Communication

4

3

and disorientation

Can speak some words irregularly,

| **Item** | **Test** | **Patient's Response**  which are understandable but  meaningless | **Score** |
| --- | --- | --- | --- |
|  | Verbal Communication  Verbal Communication | Makes sounds, but the sounds cannot be  understood  Makes no sound (no verbal response) | 2  1 |

Total Score: ________

Assessor: ________ Assessment Date: ________

Remarks:GCS = E Score + M Score + V Score; The minimum score is 3, and the maximum score is 15.

Score ≤ 8: Indicates coma

Score ≥ 9: Indicates no coma Score < 8: Severe injury

Score 9–11: Moderate injury Score ≥ 12: Mild injury

**Glasgow Outcome Scale (GOS)**

Name: ________ Gender: ____ Age: ____ Department & Bed Number: ________

Hospitalization Number: ________ Diagnosis:

| **Score** | **Grade** | **Patient's Response** | **Descriptive Evaluation** |
| --- | --- | --- | --- |
| 5 | Good  Recovery | Returns to normal life, may  have mild neurological and  psychological deficits | Returning to work is an  unrealistic rehabilitation  indicator; instead, it should  include indicators of social  activities such as leisure  activities and family  relationships. |
| 4 | Moderate  Disability | Disabled but able to live  independently (disabilities  include language impairment,  mild hemiplegia, ataxia,  intellectual and memory  deficits, personality changes,  etc.) | Independence is more  important than activities of  daily living. Able to work in a  protected environment and use  public transportation. |
| 3 | Severe  Disability | Conscious but disabled;  requires assistance with daily  living | Needs care due to physical and/or mental disabilities. |
| 2 | Vegetative  State | Only minimal responses; eyes  can open with sleep-wake  cycles | Although the cortical structure  may be intact, cerebral cortex  function is lost. |
| 1 | Death | Death | A distinction should be made  between death caused by  primary brain injury and death  caused by complications. |
